# Impact of Postoperative Chemotherapy in Patients with Gastric/Gastroesophageal Adenocarcinoma Treated with Perioperative Chemotherapy

**DOI:** 10.3390/curroncol29030161

**Published:** 2022-03-14

**Authors:** Alexej Ballhausen, Prisca Bartels, Ines Iacovella, Anica Hoegner, Alessandro Lorusso, Dmitry Bichev, Severin Daum, Peter Thuss-Patience

**Affiliations:** 1 Department of Hematology, Oncology and Tumor Immunology, Charité—Universitätsmedizin Berlin, 13353 Berlin, Germany; alexej.ballhausen@charite.de (A.B.); ines-i@hotmail.it (I.I.); anica.hoegner@charite.de (A.H.); alessandro.lorusso@charite.de (A.L.); peter.thuss@charite.de (P.T.-P.); 2 GLG Werner Forßmann Klinikum Eberswalde, 16225 Eberswalde, Germany; dmitry.bichev@klinikum-barnim.de; 3 Department of Gastroenterology, Infectiology and Rheumatology, Charité—Universitätsmedizin Berlin, 10117 Berlin, Germany; severin.daum@charite.de

**Keywords:** gastroesophageal cancer, perioperative chemotherapy, postoperative chemotherapy

## Abstract

Perioperative chemotherapy is the standard of care for patients undergoing curative resection for gastroesophageal adenocarcinoma. However, less than 50% of patients complete postoperative chemotherapy, and the added benefit to preoperative chemotherapy remains unclear. The aim of this study was to compare disease-free and overall survival (DFS and OS) in patients with perioperative chemotherapy to those who received preoperative chemotherapy only. In addition, a current literature overview is included. This multicenter, retrospective case series included 124 patients with gastroesophageal adenocarcinoma undergoing potentially curative resection and receiving pre- or perioperative chemotherapy between 2006 and 2010. Histopathological, demographic, clinical, and survival data were used to identify the impact of perioperative vs. preoperative chemotherapy on DFS and OS. Patients with perioperative chemotherapy had significantly improved DFS and OS (median DFS 28.0 months; 95%CI 0–62.4 vs. 19.0 months; 95%CI 10.5–27.5; *p* = 0.008 and median OS 35.7 months; 95%CI 0–73.6 vs. 19.2 months; 95%CI 7.8–30.4; *p* = 0.002). However, in contrast to patients with tumor-free lymph nodes at the time of resection, patients with positive lymph node status did not significantly benefit from additional postoperative chemotherapy in subgroup analysis. Further studies are encouraged to investigate optimal adjuvant treatment strategies for primary chemotherapy-resistant patients.

## 1. Introduction

Perioperative chemotherapy is the current standard of care in Europe for localized adenocarcinoma of the stomach, the gastroesophageal junction, and the distal esophagus since results from the MAGIC trial and FNLCC ACCORD07-FFCD 970363 proved significant improvement in overall survival (OS) for patients treated with perioperative chemotherapy compared to surgery alone [1,2,3,4]. In addition, perioperative chemotherapy did not increase morbidity from surgery. The German FLOT-4 trial compared the FLOT regimen (5-FU, leucovorin, oxaliplatin, docetaxel) with ECX/ECF (epirubicin, cisplatin, capecitabine/5-FU) and showed a significant improvement in OS for FLOT in 716 patients (median OS 35 months with ECX/ECF vs. 50 months with FLOT, *p* = 0.012), making perioperative chemotherapy with the FLOT regimen the current standard of care in this setting [4]. However, completion of postoperative chemotherapy is challenging. Previous data suggest that less than 50% of patients in a perioperative treatment concept actually receive the entire course of postoperative chemotherapy and the benefit it adds to preoperative chemotherapy remains unclear. To date, there has been no prospective data comparing preoperative chemotherapy alone with perioperative chemotherapy.

The aim of this study was to compare patients who received preoperative and postoperative chemotherapy with patients who only received preoperative chemotherapy in terms of disease-free survival (DFS) and OS. In addition, clinical parameters such as age at diagnosis, gender, tumor stage (T and N status at baseline and resection), postoperative complications, chemotherapy regimens, were analyzed for impact on DFS and OS in the respective subgroups. Finally, a comprehensive literature review was conducted summarizing existing evidence from previous studies for the role of postoperative chemotherapy.

## 2. Patients and Methods

### 2.1. Study Design and Objectives

This was a multicenter, retrospective case series of 124 patients diagnosed with nonmetastatic gastroesophageal adenocarcinoma between 2006 and 2010 at our center (Charité—Universitätsmedizin Berlin) or participating centers of the AIO-STO-DCX trial [5]. Electronic medical records from two participating departments at Charité—Universitätsmedizin Berlin (Department of Hematology and Oncology, Campus Virchow-Klinikum, and Department of Gastroenterology, Campus Benjamin-Franklin) were searched for patients diagnosed with nonmetastatic gastroesophageal adenocarcinoma undergoing perioperative chemotherapy with curative intent. Patients starting a preoperative (MAGIC-like) ECF/ECX-based chemotherapy with curative intent between 2006 and 2010 were included. In addition, after 2008, patients participating in the multicenter AIO-STO-DCX trial treated with perioperative DCF/DCX-based chemotherapy were included. Patient age at diagnosis, gender, tumor stage (T and N status at baseline and resection), postoperative complications, chemotherapy regimens, DFS, and OS were extracted and calculated from electronic medical records (for 61 patients from retrospective, monocentric case review) or electronic case report forms (for 49 patients from the multicenter AIO-STO-DCX trial). Local recurrence and distant metastatic disease were detected according to local standards and national guidelines or according to study protocol (for patients treated within AIO-STO-DCX trial) with scheduled clinical visits every 3 months, imaging every 3 months (alternating CT-scan with chest-XR/abdominal ultrasound), and endoscopy every 6 months.

### 2.2. Statistical Analyses

Patient demographics, treatment, and clinical features were summarized using mean, median, standard deviation, and minimum/maximum values for continuous variables and *n* (%) for categorical/ordinal variables. Patients were followed from the initial presentation date for primary gastroesophageal adenocarcinoma until local recurrence or development of metastatic disease for DFS, and from presentation until death from any cause for OS. Patients not experiencing an outcome were censored at the date of the last follow-up for local and distant recurrence and for OS. DFS and OS were estimated using the Kaplan–Meier method, and differences between strata based on clinical characteristics were assessed using log-rank tests. All tests were 2-sided, and *p*-values < 0.05 were considered statistically significant. Statistical analyses were performed using the SPSS 27 (SPSS, Chicago, IL, USA) software program.

### 2.3. Review of the Literature

Similar studies included in the literature review were identified from the MEDLINE database using the PubMed search engine without language restrictions and publication date between 2013 and 2021. The following search terms were used: “gastric cancer” OR “gastroesophageal cancer” AND “chemotherapy” AND “perioperative” OR “postoperative” OR “adjuvant” AND “preoperative” OR “neoadjuvant”. According to the scope of our study, search results were filtered for original studies comparing perioperative vs. preoperative chemotherapy alone with reported survival data from either the whole cohort or subgroup analyses. A total of 12 studies were identified to meet these criteria.

## 3. Results

### 3.1. Patient Characteristics

**Figure 1:**
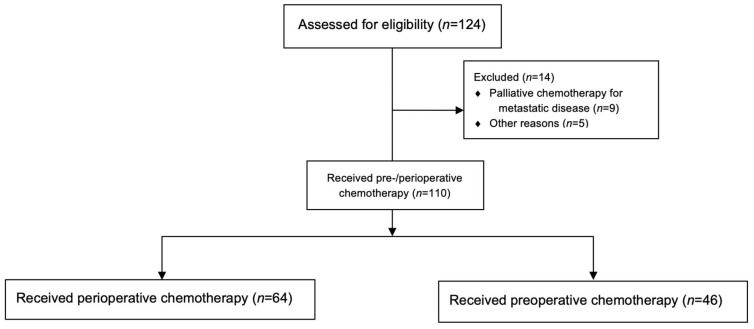
CONSORT flow diagram.

Of 124 patients with localized adenocarcinoma of the stomach or gastroesophageal junction assessed, 9 had tumor progression and went on to receive palliative chemotherapy, and for 5 patients, information on perioperative chemotherapy was missing (Figure 1). Of 110 patients included in the study, 20 (18.2%) were female and 90 (81.2%) male. The median age at diagnosis was 65 years (range 32–79). The median follow-up was 61.9 months. ECOG performance status, primary tumor localization and histology, type of chemotherapy received, T and N status at baseline and time of resection, for all patients and by completion of perioperative chemotherapy status are summarized in Table 1. Patients were assigned to the perioperative chemotherapy group if they received at least one cycle of postoperative chemotherapy.

A total of 46 patients (41.8%) did not receive postoperative chemotherapy, mainly due to postoperative complications and morbidity. Notably, patients who received perioperative chemotherapy were significantly more likely to be male (58 of 64, 90.6%), compared with patients who received preoperative chemotherapy alone (32 of 46, 69.6%; *p* = 0.005, Table 1). In addition, patients who received perioperative chemotherapy were significantly more likely to receive DCX regimen (docetaxel 75 mg/m^2^ plus cisplatin 60 mg/m^2^ (day 1), followed by oral capecitabine 1875 mg/m^2^, divided into two-dose therapy (days 1–14; every 3 weeks) in the AIO-STO-DCX trial (37 of 64, 57.8%), compared with patients who received only preoperative chemotherapy (12 of 64, 26.1%; *p* < 0.001, Table 1). Notably, distribution of T and N status at the time of resection, as well as Becker tumor regression grades of patients receiving perioperative vs. preoperative chemotherapy alone, showed significant differences (Table 1). In contrast, there were no significant differences within both groups in terms of age at initial diagnosis, ECOG performance status, primary tumor location, histology, and T and N status at baseline.

### 3.2. Outcomes

#### 3.2.1. Disease-Free and Overall Survival

**Figure 2:**
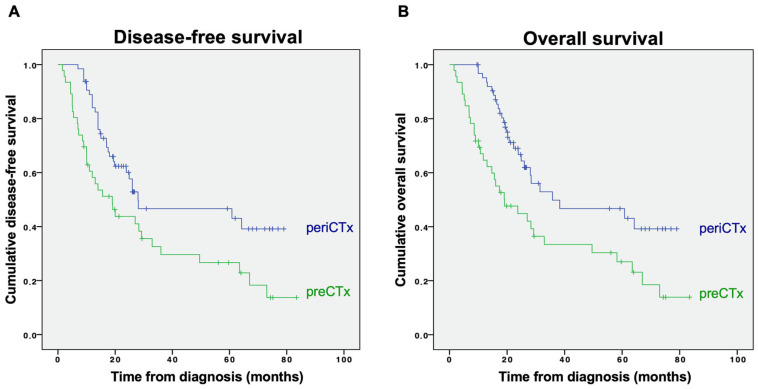
(**A**) Disease-free and (**B**) overall survival for patients receiving perioperative (periCTx, blue line) and preoperative chemotherapy (preCTx, green line) is displayed.

Patients who received perioperative chemotherapy had significantly longer DFS (median DFS 28.0 months; 95%CI 0–62.4), compared with patients receiving only preoperative chemotherapy (median DFS 19.0 months; 95%CI 10.5–27.5; *p* = 0.008; Figure 2A).

Similarly, patients who received perioperative chemotherapy had significantly longer OS (median OS 35.7 months; 95%CI 0–73.6), compared with patients receiving only preoperative chemotherapy (median OS 19.2 months; 95%CI 7.8–30.4; *p* = 0.002; Figure 2B). Notably, no significant difference in OS was found between patients receiving ECF/ECX or DCF chemotherapy regimens (median OS 27.0, 95%CI 8.7–45.3 vs. 28.1 months, 95%CI 22.9–33.3; *p* = 0.592).

#### 3.2.2. Tumor-Specific Survival

**Figure 3:**
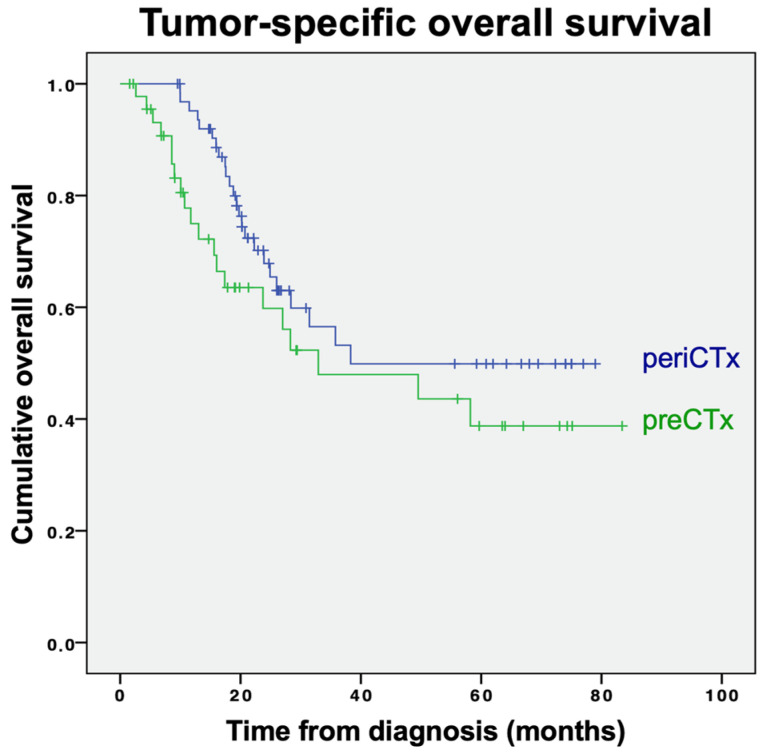
Tumor-specific overall survival for patients receiving perioperative (periCTx, blue line) or preoperative chemotherapy (preCTx, green line).

To account for postoperative complications and other causes of mortality, we defined tumor-specific survival by censoring patients whose death was not tumor-related. In contrast to our previous analysis, there was no significant difference in tumor-specific survival between patients who received perioperative chemotherapy and patients who received preoperative chemotherapy only (*p* = 0.145; Figure 3).

#### 3.2.3. Completeness of Postoperative Chemotherapy

**Figure 4:**
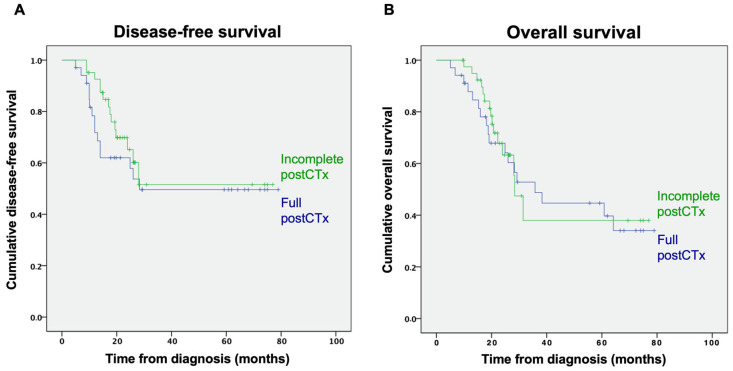
(**A**) Disease-free and (**B**) overall survival by completeness of postoperative chemotherapy (postCTx). Full postCTx status is represented by blue lines. Green lines represent incomplete postCTx status.

Of 64 patients receiving postoperative chemotherapy, 36 patients (56.3%) completed all cycles, while 28 patients (43.8%) did not complete postoperative therapy. In addition, 30 patients (46.9%) had dose reductions of their postoperative chemotherapy, while 34 (53.2%) did not. Notably, patients who received all cycles of postoperative chemotherapy as planned did not have improved DFS or OS, compared with those who had dose reductions or did not receive all cycles of postoperative chemotherapy (median DFS not reached; *p* = 0.391 and median OS 35.8; 95%CI 20.2–51.4; vs. 28.4 months; 95%CI 23.9–32.8; *p* = 0.741; Figure 4).

#### 3.2.4. Lymph Node Involvement at Baseline and at Time of Resection

**Figure 5:**
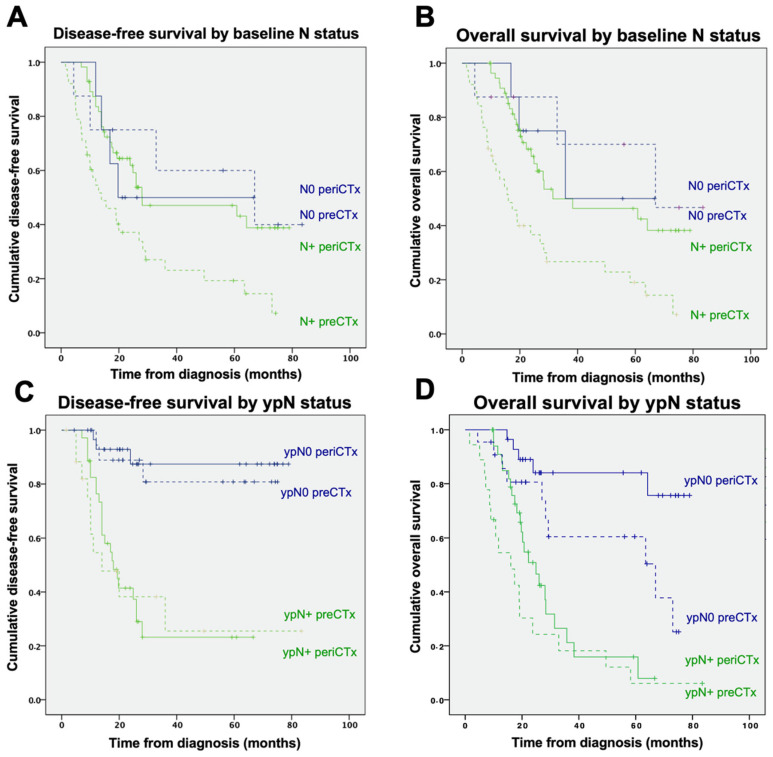
Disease-free (DFS) and overall survival (OS) by nodal status at baseline (DFS (**A**); OS (**B**)) and by at time of resection (DFS (**C**); OS (**D**)). Negative nodal status is represented by blue lines. Green lines represent positive nodal status. Survival of patients receiving preoperative chemotherapy (preCTx) is marked by dashed lines.

At baseline, patients with lymph node involvement had significantly longer DFS (Figure 5A) and OS (Figure 5B) when they received perioperative chemotherapy, compared with patients with lymph node involvement at baseline, who received only preoperative chemotherapy (median DFS 28.0 months; 95%CI 0–62.4 vs. 14.0 months; 95%CI 4.9–23.0; *p* = 0.001 and median OS 31.4 months; 95%CI vs. 16.0 months; 95%CI; *p* < 0.001).

In contrast, for patients without lymph node involvement at baseline, there was no significant impact of perioperative vs. preoperative chemotherapy on DFS and OS (median DFS 19.7 months; 95%CI; vs. 34 months; 95%CI 0–134; *p* = 0.589 and median OS 35.77 months; 95%CI; vs. 67 months; 95%CI; *p* = 0.623).

At the time of surgical resection, patients without pathological evidence of lymph node metastases (ypN0) receiving perioperative chemotherapy had a significant improvement in OS, compared with those with preoperative chemotherapy alone (median OS not reached vs. 67 months, 95%CI 18.3–115.7; *p* = 0.026, Figure 5D).

In contrast, patients who had lymph node involvement at the time of resection showed no significant improvement of DFS (Figure 5C) or OS (Figure 5D) with the addition of postoperative chemotherapy (median DFS 18 months; 95%CI 12.2–23.7; vs. 10 months; 95%CI 7.9–12; *p* = 0.062 and median OS 24.9 months; 95%CI 18.24–31.55; vs. 16 months; 95%CI 7.3–24.7; *p* = 0.137).

### 3.3. Review of the Literature

To summarize previous data and to put our data into perspective, we performed a systematic review of existing studies (Table 2) and summarized subgroup analyses of the respective studies focusing on the potential benefit of postoperative chemotherapy (Table 3).

## 4. Discussion

In this study, we investigated the role of postoperative chemotherapy analyzing 110 patients from our center and the AIO-STO-DCX trial [5]. We showed that patients who continued perioperative chemotherapy postoperatively had a significantly better DFS and OS, compared with patients receiving preoperative chemotherapy alone. Median survival was increased by 16.5 months (*p* = 0.002).

As there are currently no data from randomized trials, we also performed a systematic review of the available studies (Table 2) [6,7,8,9,10,11,12,13,14,15,16,17]. We identified a total of 12 retrospective studies, most of them with a relatively small sample size from single European centers. Overall, 3 studies with a larger sample size including between 1600 and 3500 patients were based on multicenter, national cancer databases from the US and the Netherlands. In summary, these studies revealed conflicting results with regard to a potential benefit of postoperative chemotherapy for resectable gastroesophageal adenocarcinomas.

In our study, we identified a significantly improved DFS and OS in patients who received pre- and postoperative chemotherapy, compared with those who received only preoperative chemotherapy. In line with this, 5 of the previous studies including one of the larger database analyses similarly found a significantly improved survival in patients with perioperative, compared with preoperative chemotherapy alone [9,10,13,15,17]. However, in most studies, administration of postoperative chemotherapy did not result in improved survival. Indeed, our analysis for tumor-specific survival, correcting for non-tumor-specific mortality, did not find a significant benefit of postoperative chemotherapy. Notably, one German study even indicated a shorter survival in patients receiving postoperative chemotherapy [14]. However, with only 72 patients, this is one of the smallest studies of our literature review. In addition to mostly small sample sizes, further potential reasons for these conflicting results include the retrospective nature and single-center designs in the majority of these studies. Moreover, study populations were relatively heterogeneous with regards to demographics, tumor stage, primary tumor location (i.e., including lower esophageal adenocarcinoma), and applied chemotherapy regimens.

Most studies did not specify the number of postoperative chemotherapy cycles administered. Luc et al. determined a minimal number of two postoperative chemotherapy cycles necessary to improve survival [16]. In contrast, in an analysis of 134 patients from Germany, Glatz et al. showed that the completion of all scheduled cycles had no significant impact on patient survival, compared with only one cycle of postoperative chemotherapy [15]. In line, we also did not find a negative impact of premature discontinuation or dose reduction in postoperative chemotherapy, compared with completion of postoperative chemotherapy with regards to DFS and OS. However, as most studies did not evaluate the impact of the number of postoperative chemotherapy cycles, a definite threshold for a minimal number of cycles necessary to improve survival remains unclear.

With mixed results from prior studies for the entire cohorts, efforts have been made to identify subgroups that may, nevertheless, benefit from postoperative chemotherapy. A systematic overview of the identified subgroups from earlier studies is provided in Table 3.

A negative lymph node status at the time of resection was the only independent predictor of OS in the perioperative arm of the MAGIC trial by multivariate analysis [18]. Correspondingly, in our study, we found that patients with the ypN0 stage receiving both pre- and postoperative chemotherapy showed a significant survival benefit, compared with preoperative chemotherapy alone, while patients with the ypN+ stage did not. Papaxoinis et al. similarly found an improved DFS in ypN0 patients with perioperative chemotherapy, compared with preoperative chemotherapy alone [8]. In contrast, a large multi-institutional US cohort showed that only gastric cancer patients with ypN1 disease benefitted from continuing postoperative chemotherapy [7]. Similarly, Glatz et al. showed that the benefit from continuing chemotherapy post-surgery might be limited to those with ypN+ tumors. Moreover, other studies did not show an association between ypN status and postoperative survival benefit [6,11]. While it remains unclear whether these “non-responders” to preoperative chemotherapy (defined as patients with ypN+) in the MAGIC trial would have had even worse outcomes with surgery alone, our results show that the addition of postoperative chemotherapy does not lead to significant benefits in DFS or OS in this “non-responder” group.

Furthermore, Deng et al. and Saunders et al. showed a survival advantage by additional postoperative chemotherapy only for patients with good histopathologic response to preoperative chemotherapy [6,12]. On the other hand, Glatz et al. identified a benefit for the subgroup of patients with poor histopathological response, defined as >50% residual tumor [15]. Finally, other identified subgroups with a benefit of postoperative chemotherapy include patients with R1 resection [8], treatment with FLOT chemotherapy, or tumors with non-intestinal histology [11].

Our study had several limitations. These include its retrospective design and the treatment with outdated ECF/DCF-like regimens, mainly due to the historic period (2006–2010) of our study cohort. Thus, the impact of treatment with currently widely used perioperative regimens (i.e., FLOT) on identified prognostic parameters remains unclear. Nevertheless, the principle of perioperative chemotherapy in gastric cancer was established by ECF. Therefore, questioning this principle in its original form may also have implications for future refinement of perioperative chemotherapy. Furthermore, investigating the DCX regimen, which consists of docetaxel/capecitabine and cisplatin, the same chemotherapy backbone (platinum/fluoropyrimidine/taxane) as in FLOT, may also suggest similar implications for FLOT. Results of our subgroup analysis are limited due to the sample size and should be considered a hypothesis-generating approach. In addition, a systematic bias toward healthier or fitter patients receiving postoperative therapy cannot be ruled out. This is underlined by the fact that the trial population of our cohort had significantly higher rates of completing perioperative chemotherapy, likely due to a selection bias toward a fitter and healthier population. Despite these limitations, we found that especially in primary chemotherapy-resistant (i.e., ypN+) tumors, the benefit of postoperative chemotherapy remains uncertain. Further studies are encouraged to evaluate optimal adjuvant treatment strategies for these patients. As such, results are awaited from the polish STOPEROPCHEM trial (NCT01787539), the first randomized controlled trial assessing the role of postoperative chemotherapy in patients with histopathological response to preoperative chemotherapy. However, this study will also be limited by the selection of the older chemotherapy regimen ECX. Importantly, currently recruiting prospective randomized trials investigate intensifying preoperative chemotherapy [19] or integrating checkpoint inhibition as postoperative treatment for patients with ypN+ or R1 tumors in the VESTIGE trial (NCT03443856) [20].

In summary, the present study was able to demonstrate a survival benefit by continuing perioperative ECX- or DCF-like chemotherapy after surgery. In addition, subgroup analysis showed a specific benefit for patients with tumor-free lymph nodes at the time of resection.

## 5. Conclusions

Overall, although our study supports the post-surgery continuation of perioperative chemotherapy, especially in chemotherapy-responsive tumors, the available evidence remains inconclusive. Ultimately, the ongoing and future multicenter prospective randomized trials will help determine the definite impact of postoperative chemotherapy in the perioperative treatment of gastroesophageal cancer.

## Figures and Tables

**Table 1:** Patient characteristics.

Variable	All Patients (*n* = 110)		Perioperative Chemotherapy ° (*n* = 64)		Preoperative Chemotherapy (*n* = 46)		*p*-Value * (Perioperative vs. Preoperative Chemotherapy)
	*n*	%	*n*	%	*n*	%	
Age at initial diagnosis in years							0.325 ^§^
Mean (standard deviation)	62.76 (10.12)		61.44 (10.01)		64.61 (10.10)		
Median (minimum, maximum)	65 (32, 79)		64 (32, 79)		67 (36, 77)		
Gender							0.005
Male	90	81.8%	58	90.6%	32	69.6%	
Female	20	18.2%	6	9.4%	14	30.4%	
ECOG performance status							0.621
0	105	95.5%	60	93.8%	45	97.8%	
1	5	4.5%	4	6.3%	1	2.2%	
Primary tumor location							0.104
Esophagus	11	10.0%	6	9.4%	5	10.9%	
Gastroesophageal junction	50	45.5%	34	53.1%	16	34.8%	
Stomach	49	44.5%	24	37.6%	25	54.4%	
Histology							0.609
Intestinal type	51	46.4%	28	43.8%	23	50.0%	
Diffuse type	36	32.7%	20	31.3%	16	34.8%	
Mixed type	10	9.1%	6	9.4%	4	8.7%	
Not specified	6	5.5%	4	6.3%	2	4.3%	
Chemotherapy							<0.001
ECF/ECX	61	55.5%	27	42.2%	34	73.9%	
DCF/DCX	49	44.5%	37	57.8%	12	26.1%	
T and N status at baseline							0.691
uT2	10	9.1%	7	10.9%	3	6.5%	0.218
uT3	92	83.6%	52	81.3%	40	87.0%	
uT4	8	7.3%	5	7.8%	3	6.5%	
uN0	16	14.5%	8	12.5%	8	17.4%	
uN1	72	65.5%	39	60.9%	33	71.7%	
uN2	1	0.9%	1	1.6%	0	0%	
uN+	21	19.1%	16	25.0%	5	10.9%	
T and N status at time of resection							<0.001
ypT0	15	13.6%	8	12.5%	7	15.2%	<0.001
ypT1	10	9.1%	7	10.9%	3	6.5%	
ypT2	51	46.4%	33	51.6%	18	39.1%	
ypT3	23	20.9%	16	25.0%	7	15.2%	
ypT4	4	3.6%	0	0%	4	8.7%	
ypN0	50	45.5%	29	45.3%	21	45.7%	
ypN1	34	30.9%	26	40.6%	8	17.4%	
ypN2	11	10.0%	8	12.5%	3	6.5%	
ypN3	8	7.3%	1	1.6%	7	15.2%	
Becker tumor regression grading							0.001
1a (complete response)	15	13.6%	8	12.5%	7	15.2%	
1b (<10% residual tumor)	8	7.3%	4	6.3%	4	8.7%	
2 (10–50% residual tumor)	33	30.0%	20	31.3%	13	28.3%	
3 (>50% residual tumor)	47	42.7%	32	50.0%	15	32.6%	
Not available	7	6.3%	0	0%	7	15.2%	

° Patients receiving at least one cycle of postoperative chemotherapy. * Chi-square for nonparametric variables. ^§^ Kolmogorov–Smirnov test.

**Table 2:** Characteristics of retrospective studies.

Study	Country	Multi-Center, *n*	Recruitment Period	Primary Tumor Location			Chemotherapy Regimen		Patients		Perioperative vs. Preoperative Chemotherapy Alone		
				Esophagus	GEJ	Stomach	Epirubicin Based	Docetaxel Based	Total *n*	periCTx *	Favors periCTx (OS)?	Reported Statistical Analysis	General Remarks
Deng et al., 2021 [6]	USA	yes, n.a. (NCDB)	2006–2017	-	-	100%	n.a.	n.a.	2382	36%	no	HR 0.88 (95%CI 0.75–1.02), *p* = 0.37	
Drake et al., 2020 [7]	USA	yes, n.a. (NCDB)	2006–2014	-	-	100%	n.a.	n.a.	3449	32%	no	median OS: 56.8 vs. 52.5 mo, *p* = 0.131; 5-year survival 48.9% vs. 47.3%	PSM applied
Papaxoinis et al., 2019 [8]	UK	yes, *n* = 3	2009–2017	33%	67%	-	99% ECX(like)	-	312	72%	no	median OS: 46.1 vs. 36.7 mo, *p* = 0.199	PSM applied; no difference in DFS (22.2 vs. 25.7 mo, *p* = 0.627) and postrelapse survival (15.3 vs. 8.7 mo, *p* = 0.122)
Coimbra et al., 2019 [9]	Brazil	no	2006–2016	-	-	100%	30% ECX(like), 59% PF	11% DCF/ DCX	225	65%	yes	5-year survival 70.3% vs. 59.9%; HR 0.55 (95%CI 0.33–0.91, *p* = 0.019)	after exclusion of patients with postoperative death, postoperative treatment did not remain as an independent predictor of survival
van Putten et al., 2019 [10]	Netherlands	yes, n.a. (NCR)	2006–2014	-	-	100%	n.a.	n.a.	1686	57%	yes	HR 0.80 (95%CI 0.70–0.93); PSM analysis: HR 0.84 (95%CI 0.71–0.99)	some of the patients received postoperative chemoradiotherapy, proportion not reported
Sisic et al., 2017 [11]	Germany	no	2006–2015	-	62%	38%	46% ECF(like), 17% others (PF/FLO/OX)	36% FLOT	299	57%	no	median OS: 78.2 mo vs. n.r., *p* = 0.331	no difference in DFS (43.3 vs. 41.1 mo, *p* = 0.118)
Saunders et al., 2017 [12]	UK	no	2006–2013	35%	47%	17%	100% ECX(like)	-	333	57%	n.a.	n.a.	statistical analysis only for subgroups reported, see Table 3
Karagkounis et al., 2017 [13]	USA	yes, *n* = 8	2000–2012	-	23%	73%	79% ECX(like)	-	163	69%	yes	HR 0.33 (95%CI 0.14–0.82), *p* = 0.01	improved DFS (HR 0.52, 95%CI 0.27–0.96)
Lichthardt et al., 2016 [14]	Germany	no	2006–2013	-	42%	57%	ECX/ECF (% n.a.)	FLO(T) (% n.a.)	72	72%	no	trend for shorter survival for periCTx, but not statistically significant (*p* = 0.101)	after exclusion of two patients with perioperative death (corresponding to all other study protocols), statistically significant shorter 3-year-survival for patients with periCTx: 71.2% vs. 100%, *p* = 0.038
Glatz et al., 2015 [15]	Germany	no	2006–2013	-	72%	28%	43% ECF/EOX	57% FLOT	134	64%	yes	med. OS: n.r. vs. 44 mo; 5-year survival 75.8% vs. 40.3%, *p* < 0.001	
Luc et al., 2015 [16]	France	no	2000–2012	18%	43%	39%	ECF (% n.a.)	DCF (% n.a.)	110	67%	no	median OS: 43 vs. 20 mo, *p* = 0.59	no difference in DFS (35 vs. 11 mo, *p* = 0.098); additional analysis identified two cycles of postCTx necessary to improve survival (HR 5.13, 95%CI 1.55–16.97, *p* = 0.007)
Mirza et al., 2013 [17]	UK	no	1996–2010	-	64%	36%	100% ECF	-	66	47%	yes	significant difference (*p* = 0.02); HR 0.26, *p* = 0.008	

GEJ, gastroesophageal junction; ECX/ECF, epirubicin, cisplatin, capecitabine/5-fluorouracil (5-FU); FLOT, 5-FU, leucovorin, oxaliplatin, docetaxel; PF, platin, fluoropyrimidine; DCF, docetaxel, cisplatin, 5-FU; * periCTx, percentage of patients with preoperative and at least one cycle of postoperative chemotherapy; OS overall survival; NCDB, US National Cancer Database; NCR, Netherlands Cancer Registry; HR, hazard ratio; CI, confidence interval; mo, months; n.r., not reached; PSM, propensity score matching; DFS disease-free survival.

**Table 3:** Identified subgroups with benefits from perioperative chemotherapy in retrospective studies.

Study	Subgroup with Benefit from periCTx	Number of Patients		Subgroup Analysis: periCTx vs. preCTx Alone
		*n*	periCTx * vs. preCTx Alone	
Deng et al., 2021 [6]	good HPR (pTNM < cTNM stage, excluding ypT0N0)	727	255 vs. 472	improved 5-year survival in periCTx patients with preCTx sensitive disease (73.8% vs. 65.0%; HR 0.64, 95%CI 0.46–0.91, *p* = 0.02); no benefit from periCTx in subgroups with (i) very sensitive disease (ypT0N0) and (ii) refractory disease (pTNM ≥ cTNM)
Drake et al., 2020 [7]	ypN1 (AJCC 8th)	678	222 vs. 456	improved OS in periCTx patients with ypN1 disease (79.6 vs. 41.3 mo; *p* = 0.025)
Papaxoinis et al., 2019 [8]	R1	104	69 vs. 35	improved OS (HR 0.53, 95%CI 0.31–0.90, *p*=0.018) and DFS (HR 0.56, 95%CI 0.33–0.94, *p* = 0.027) in periCTx patients with R1 resection
	ypN0	129	94 vs. 35	improved DFS in periCTx patients with tumor-free lymph nodes (HR 0.35, 95%CI 0.13–0.95, *p* = 0.038); trend for improved OS (HR 0.44; 95%CI 0.19–1.0, *p* = 0.051)
Sisic et al., 2017 [11]	FLOT	108	74 vs. 34	improved DFS in periCTx patients receiving FLOT regimen (n.r. vs. 37.7 mo, *p* = 0.038)
	nonintestinal tumors	111	65 vs. 46	improved DFS in periCTx patients with nonintestinal tumors (56.2 vs. 20.3 mo, *p* = 0.023)
Saunders et al., 2017 [12]	good HPR (TRG 1–3)	129	70 vs. 59	improved OS in periCTx patients with preCTx responsive disease (HR 0.51, 95%CI 0.28–0.93, *p* = 0.028)
Karagkounis et al., 2017 [13]	stage II (AJCC 7th)	43	26 vs. 17	improved DFS in periCTx patients with stage II tumors (20% vs. 64.7%, *p* = 0.003)
Glatz et al., 2015 [15]	ypN+	56	33 vs. 23	improved 5-year survival in periCTx patients with ypN+ stages (64.5% vs. 9.7%, *p* = 0.002)
	poor HPR (>50% vital tumor cells)	64	36 vs. 28	improved 5-year survival in periCTx patients with poor HPR (55.5% vs. 19.3%, *p* = 0.015)

* periCTx, perioperative chemotherapy, defined as preoperative chemotherapy and at least one cycle of postoperative chemotherapy received; preCTx, preoperative chemotherapy; HPR, histopathological response to preoperative chemotherapy; AJCC, American Joint Committee on Cancer; OS, overall survival; mo, months; n.r., not reached; FLOT, 5-FU, leucovorin, oxaliplatin, docetaxel; TRG, Mandard tumor regression grades.

## Data Availability

The data presented in this study are available on request from the corresponding author.
